# Unexpected genetic diversity of *Mycoplasma agalactiae* caprine isolates from an endemic geographically restricted area of Spain

**DOI:** 10.1186/1746-6148-8-146

**Published:** 2012-08-27

**Authors:** Christian De la Fe, Joaquín Amores, Florence Tardy, Eveline Sagne, Laurent-Xavier Nouvel, Christine Citti

**Affiliations:** 1 Departamento de Sanidad Animal, Facultad de Veterinaria, Universidad de Murcia, Campus de Espinardo s/n, 30100, Murcia, Spain; 2 UMR Mycoplasmoses des Ruminants, Anses, Laboratoire de Lyon, 31 Avenue Tony Garnier, 69364, Lyon Cedex 07, France; 3 INRA, UMR 1225, Ecole Nationale Vétérinaire de Toulouse, 23 Chemin des Capelles, 31076, Toulouse Cedex 3, France; 4 Université de Toulouse, INP-ENVT, UMR 1225, Ecole Nationale Vétérinaire de Toulouse, 23 Chemin des Capelles, 31076, Toulouse Cedex 3, France

**Keywords:** *Mycoplasma agalactiae*, Molecular typing, Contagious agalactia, Goats

## Abstract

**Background:**

The genetic diversity of *Mycoplasma agalactiae* (MA) isolates collected in Spain from goats in an area with contagious agalactia (CA) was assessed using a set of validated and new molecular typing methods. Validated methods included pulsed field gel electrophoresis (PFGE), variable number of tandem repeats (VNTR) typing, and Southern blot hybridization using a set of MA DNA probes, including those for typing the *vpma* genes repertoire. New approaches were based on PCR and targeted genomic regions that diverged between strains as defined by *in silico* genomic comparisons of sequenced MA genomes.

**Results:**

Overall, the data showed that all typing tools yielded consistent results, with the VNTR analyses being the most rapid method to differentiate the MA isolates with a discriminatory ability comparable to that of PFGE and of a set of new PCR assays. All molecular typing approaches indicated that the Spanish isolates from the endemic area in Murcia were very diverse, with different clonal isolates probably restricted to separate, but geographically close, local areas.

**Conclusions:**

The important genetic diversity of MA observed in infected goats from Spain contrasts with the overall homogeneity of the genomic background encountered in MA from sheep with CA in Southern France or Italy, suggesting that assessment of the disease status in endemic areas may require different approaches in sheep and in goats. A number of congruent sub-typing tools are now available for the differentiation of caprine isolates with comparable discriminatory powers.

## Background

Contagious agalactia (CA) is a disease of small ruminants associated with several clinical signs, including mastitis, arthritis, keratoconjunctivitis, pneumonia and septicaemia. CA is listed by the World Organization for Animal Health and is responsible for significant economic loss, with its severity and expression being dependent on several factors, including the host species, the aetiological agent, the production system and the environmental conditions [1-4]. In this context, defining the sanitary status of herds or regions with respect to CA is essential, but also very challenging. Three statuses are currently recognised: disease-free areas, areas with sporadic acute outbreaks and endemic areas, where infection is widespread although not always acute [1,4]. In CA endemic regions animals typically have no to transitory clinical sign and there are many asymptomatic auricular carriers [5-8]. Preventive and therapeutic strategies remain very inefficient in the control of CA, most likely because of both pathogen-specific features and the lack of epidemiological data. Thus far management strategies have been the most satisfactory method for controlling this disease [4].

Mycoplasma species responsible for CA include *Mycoplasma mycoides* subspecies *capri* (*Mmc*), *Mycoplasma capricolum* subspecies *capricolum* (*Mcc)* and *Mycoplasma putrefaciens*, from the spiroplasma phylogenetic group, and *Mycoplasma agalactiae* (MA), from the hominis phylogenetic group, with this latter species being designated the *stricto sensu* aetiological agent of CA [1,4]. Interestingly, CA in sheep is often associated only with MA, while the situation can be more complex in goats, in which CA is commonly associated with both MA and mycoplasmas of the “mycoides” cluster [1,4,8].

The complex clinical situation described above requires tools capable of exploring the dynamics of infection in order to set up management strategies. Thus far, several typing techniques have been used to characterize CA agents, including analysis of variable numbers of tandem repeats (VNTR) [9], multilocus sequence typing (MLST) [10], Southern blots using DNA probes [11], insertion sequence (IS) typing [8,12] and pulsed field gel electrophoresis (PFGE) [8,13,14]. However, their capacity to answer epidemiological questions is variable. For instance, PFGE is very useful when comparing an asymptomatic goat herd infected with multiple polymorphic *Mmc* strains to an outbreak in which one unique clone was predominant [8]. However, application of the same technique to MA isolates in another situation resulted in poor discrimination and was not informative about the strain circulation [13].

A number of studies has been conducted to characterize and compare isolates of MA from outbreaks of CA. These have generally focused on sets of isolates from various geographical origins [11,12,14], but the reports have provided few details about the status of the herds with respect to infection, the type of herds or the animals from which the isolates were collected [9,10]. In addition, typing analyses that have been performed in well-documented epidemiological contexts have mainly examined CA in sheep [13,15] leaving the situation in goats unexplored. This gap is surprising, as CA in goats can be of considerable economical importance, and also because the two currently available genomic sequences [16,17] from which many typing tools were derived, are both from strains with a caprine origin.

The present study is, to our knowledge, the first to conduct a detailed characterization of potential molecular differences between caprine MA isolates collected in a geographically restricted area (Murcia, Spain). This area hosts a native goat breed, the Murciano Granadina, and uses a semi-intensive production system based around a single insemination centre. Although this limits the entry of animals from other regions, Murcia is known to have had endemic CA for many years [18,19]. This has raised the question of the mode of dissemination and the origins of the agent responsible for CA in this area.

The purpose of the present study was to assess the relevance of typing approaches already validated on large and diverse strain collections (PFGE, VNTR, IS-typing) to discriminate a limited number of caprine isolates from a common geographical origin (Murcia) and to explore the discriminative power of new, more specific markers. This is an essential step before addressing more complex epidemiological questions that can be crucial in improving management of CA in goats in endemic areas.

## Results

### Strain typing by PFGE

**Figure 1:**
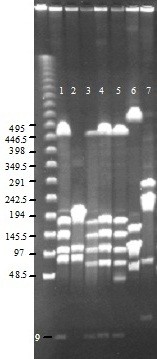
**PFGE showing representative *Sma*I profiles.** Lanes 1 and 2: Reference strains PG2 and 5632, respectively; lanes 3 and 4: isolates AG4 and AG28 (profile A); lane 5: isolate AG18 (profile A1); lane 6: isolate AG26 (profile B); lane 7: isolate AG14 (profile C). Molecular sizes (λ ladder, Bio-Rad) are indicated on the left in kb. The lowest, detectable, PG2 band (Lane1) is predicted *in silico* to be of approximately 9 kpb.

Four restriction endonucleases, *Sma*I, *Mlu*I, *Kpn*I and *Bst*ZI, were selected that yielded easily interpretable PFGE banding patterns on our set of isolates. Their profiles clustered the 13 field isolates in 3 main groups A, B, C (Table 1), as illustrated by a representative gel in Figure 1. Group A included 6 of the 13 isolates, as well as the PG2 type strain. This group was very similar to the main European MA profile reported in 2008 by McAuliffe *et al.*[9]. Interestingly, one isolate (AG18) yielded a pattern that differed slightly from that of the other members of the group. After *Sma*I digestion, it yielded the 6 characteristic fragments as well as a smaller one of 45 kb (Figure 1). Analysis with other endonucleases confirmed that this isolate appeared to have a genome that was larger than the other group A isolates by 40 to 50 kb. Group B included all isolates from bulk tank milk (BTM) samples collected in Jumilla between the months of January and March 2000. These isolates appeared to be strictly clonal. Group C included two isolates from semen that were collected in 2008 from two different goat males in Lorca. No field strain had the same restriction pattern as the sequenced 5632 strain.

**Table 1:** **Molecular typing of the *Mycoplasma agalactiae *isolates and strains**

**Isolate**	**Source**	**Origin-Date**^**a**^	**PFGE profile**^**b**^	**VNTR 5**^**c**^	**VNTR 14**^**c**^	**VNTR 17**^**c**^	**VNTR 19**^**c**^	**All VNTR profiles**^**d**^	***vpma*profiles**^**e**^	**AGA/BOV profile**^**f**^	**PCR control set**	**PCR set 1**^**g**^	**PCR set 2**^**g**^	**PCR set 3**	**PCR set 4**	**PCR set 5**	**PCR set 6**
PG2	Unk	Unk-1952	A	2.1*	2*	2.6*	4.9*	TR1	VP1a	AGB1	+	PG2	PG2	-	-	-	+
AG30	BTM	YE-2000	A	2.1	2	4.4*	4.9	TR2	VP2	AGB1	+	PG2	PG2	-	-	-	+
AG33	BTM	YE-2000	A	2.1	2	4.4	4.9	TR2	VP2	AGB1	+	PG2	PG2	-	-	-	+
AG35	BTM	YE-2000	A	2.1	2	4.4	4.9	TR2	VP2	AGB1	+	PG2	PG2	-	-	-	+
AG28	BTM	YE-2000	A	2.1*	2	4.4	4.9	TR2	VP2	AGB1	+	PG2	PG2	-	-	-	+
AG18	Mastitis	MA-2008	A1	2.1	2	4.4	4.9	TR2	VP1b	AGB1	+	PG2	PG2	-	-	-	+
AG4	BTM	LO-2009	A	2.1	2*	4.4	4.9	TR2	VP2	AGB1	+	PG2	PG2	-	-	+	+
AG13	Semen	LO-2008	C	2.1*	0	0	4.9	TR3	VP3a	AGB2	+	PG2	PG2	-	+	+	-
AG14	Semen	LO-2008	C	2.1	0	0	4.9*	TR3	VP3b	AGB2	+	PG2	PG2	-	+	+	-
AG26	BTM	JU-2000	B	2.1	0	6.3	4.9	TR4	VP5	AGB3	+	PG2	5632	+	+	+	-
AG27	BTM	JU-2000	B	2.1	0	6.3	4.9	TR4	VP5	AGB3	+	PG2	5632	+	+	+	-
AG29	BTM	JU-2000	B	2.1	0	6.3	4.9	TR4	VP5	AGB3	+	PG2	5632	+	+	+	-
AG32	BTM	JU-2000	B	2.1	0	6.3*	4.9	TR4	VP5	AGB3	+	PG2	5632	+	+	+	-
AG34	BTM	JU-2000	B	2.1	0	6.3	4.9	TR4	VP5	AGB3	+	PG2	5632	+	+	+	-
5632	Arthritis	Unk- < 1991	D	2.1	0	6.4*	4.9	TR5	VP7	AGB4	+	5632	5632	+	+	+	-

^a^Code for the geographical origin of the isolate and the year of isolation: YE: Yecla, MA: Málaga, LO: Lorca, MU: Murcia, JU: Jumilla. The symbol < is used when the exact year is unknown (Unk).

^b^Profiles as defined by PFGE analyses.

^c^Number of repeats based on the consensus sequence determined for each VNTR. * indicates VNTR for which the PCR product was sequenced.

^d^Profiles as defined by VNTR analyses based on data a combination of data generated with each VNTR.

^e^*vpma* profiles defined based on data in Additional file 2: Table S2.

^f^AGA-BOV profiles defined based on data in Additional file 2: Table S2.

^g^PG2: data obtained were identical to those obtained with strain PG2; 5632: data obtained were identical to those obtained with strain 5632.

*BTM* Bulk tank milk.

Previous PFGE analyses conducted on MA proved to discriminate poorly between local isolates from Italy [13] and, more recently, between isolates from all over Europe, all of each were found to be very similar to PG2 [9]. However, in the present study PFGE pulsotypes were not homogeneous, despite the restricted sampling area, and the three groups of strains appeared to be distinct with a discrimination index (D) of 0.73 according to Hunter and Gaston [20].

### Correlation between PFGE profiles and VNTR analyses

**Figure 2:**
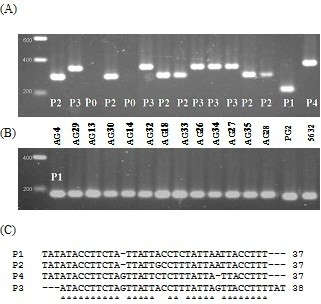
**Profiles obtained with (A) VNTR 17, (B) VNTR 5 and (C) VNTR 17 nucleotide repeat sequences corresponding to PCR products of individual profile (P0 to P4).** Positions with similar nucleotides in all profiles are marked with an asterisk. Isolates are indicated above the first gel. Lane 1, size marker (Smart Ladder Eurogentec).

Strains were subjected to VNTR analysis targeting 4 VNTRs, designated VNTR5, 14, 17, and 19, as previously described [9]. PCR results were first analysed by gel electrophoresis, which detected no difference between strains for VNTR5 or VNTR19, with all profiles identical, including those of the PG2 type strain and of 5632. While VNTR14 was of limited value as it only separated the strains into two groups, results obtained with VNTR 17 divided the 13 field isolates into 3 main clusters (TR2, TR3 and TR4) that matched the clusters obtained when comparing PFGE profiles (Table 1). These data were further supported by comparing sequences of the PCR products obtained from representatives of the different VNTR profiles (Table 2). For this purpose, PCR products from (i) VNTR 5 of isolates AG13, AG28 and PG2, (ii) VNTR 14 of isolates AG4 and PG2 (iii) VNTR 17 of PG2 (P1), AG30 (P2), AG32 (P3) and 5632 (P4) (Figure 2), and (iv) VNTR 19 of AG14 and PG2 were directly sequenced (see Additional file 1: Table S1 for each VNTR sequence). Based on these sequence data, a consensus sequence was generated (Figure 2C for VNTR17) and the number of tandem repeats defined for each VNTR (Table 2). A VNTR profile, TR1 to TR5 (Table 1), was ascribed to each strain and the overall VNTR analysis found to match the PFGE clustering, although it did not discriminate strain AG18, which had a pulsotype slightly different from the overall group A. This is reflected by the index of discrimination which is slightly lower for VNTR typing (0.67) than for PFGE (0.73).

**Table 2:** **Features of VNTRs *Mycoplasma agalactiae *isolates**

**VNTR**	**Unit length (in nt)**	**Isolate**	**Copy number**	**Size (in nt)**	**Consensus sequence**	**Percentage of identity**^**a**^
5	21	PG2	2.1	226	AAAGAATATGA	80-100%
		AG13	2.1	226	TAAAATAAAA	
		AG28	2.1	228		
14	13	PG2	2	158	TTTAGATTGCTAA	100%
		AG4	2	158		
17	37	PG2	2.6	209	TATATACCTT	86-100%
		AG30	4.4	285	CTATTATTAC	
		AG32	6.3	337	CTCTATTAA	
		5632	6.4	348	TTACCTTT	
19	21	PG2	4.9	184	TGTTTTCTTGC	80-100%
		AG14	4.9	186	TTCTTCTTGT	

^a^Proportion of sequence conserved between the strains examined.

### Using the *vpma* profiles as a typing tool

In MA, the *vpma* locus has been thoroughly studied and shown to encode genes with related sequences [15,21-23]. The size of the *vpma* repertoire (number of *vpma* genes) varies between strains, but is constant within each strain. Here, we assessed the *vpma* profiles of our set of strains by Southern blot analyses using genomic DNA digested with *Ase*I or *Alw*I and hybridised to different probes. The first probe was a single digoxigenin-labelled oligonucleotide probe, A3F, that detects the conserved 5Â´ ends of all *vpma* genes and thus assesses (when appropriate restriction endonucleases are used) the number of *vpma* genes in the strain, regardless of their specific *vpma* sequences. In contrast, the other 6 probes individually target each *vpma* gene found in the PG2 type strain (*vpmaV* to *vpmaZ*). Based on the hybridization results, a *vpma* profile was assigned to each strain (Table 1 and Additional file 2: Table S2 for detailed results).

As shown in Table 1, the *vpma* profiles were slightly more discriminative (D = 0.74) than the VNTR or PFGE profiles because they divided the 13 isolates into 3 main groups, and differentiated AG18, AG13 and AG14 into different *vpma* subtypes. Strains of pulsotype A had *vpma* profiles similar to that of PG2 except for an additional hybridizing band in A3F that suggested the presence of an additional *vpma* gene in the Spanish strains in this group. All but AG18 also had a size difference in the fragment hybridizing to *vpmaU* compared to PG2. Strains in group B, as typed by PFGE, had an identical *vpma* profile that was clearly different from those of the other groups and from PG2 and 5632. Finally, the two strains isolated from semen (AG13 and 14) were also clustered using this approach and were clearly distinct from the other groups with a number of *vpma* differences, including the absence of bands hybridizing to *vpma*U, V, Y or Z specific probes, together with a set of additional fragments hybridizing to the A3F probe. This suggested that isolates from semen possessed a different *vpma* repertoire from strains isolated from milk. These data correlated with those obtained with PFGE and VNTR.

### Identification of potential new molecular typing tools

Based on the results obtained above, we assessed whether we could further divide the 3 main groups of Spanish isolates into subtypes. For this purpose, Southern blot analyses were first conducted using a set of 7 probes designated AGA/BOV that had been identified previously as specific for MA or *Mycoplasma bovis* but that had also been found to be subject to inter-strain variation within a species (Additional file 2: Table S2). AGA/BOV profiles (Table 1) correlated perfectly with the typing clusters obtained by PFGE and VNTR analyses, but had no additional power of discrimination (D = 0.67).

Previous *in silico* comparative analysis indicated that 5632 and PG2 genomes differ by a number of genes, most of which fall into two categories: genes that are related to mobile genetic elements such as insertion sequences (IS) or integrative conjugative elements (ICE), and genes that encode enzymes of restriction modification systems [17]. Based on these data, PCR assays were then designed that either targeted the presence of a particular gene or the genomic location of particular elements (see Additional file 3: Table S3 and Additional file 4: Figure S1). A total of 20 PCR assays were performed on our set of isolates, and those with identical results were grouped (Table 1). PCR set 3 contained 6 PCR assays that yielded identical results with the isolates: no amplification from isolates in the A and C pulsotypes and amplification of a fragment of the expected size with isolates in the B and D pulsotypes. Since the assays in PCR set 3 mainly targeted mobile genetic elements such as IS or ICE, these data indicate that isolates from Yecla, Málaga and Lorca lack these mobile elements, clearly setting the Jumilla strains apart. Southern blot analyses further demonstrated the presence of the ICE only in the Jumilla isolates. The results obtained with PCR sets 3 and 6 are consistent with the grouping of the 13 isolates into 3 clusters, as with PFGE, VNTR and hybridization with AGA/BOV probes assays. Isolate AG4 could be distinguished from the others with PCR set 5 (4 PCR assays). When combined, the PCR sets (or at least a combination of 3: set 5 with set 2 or 3 and set 4 or 6) were as discriminative as PFGE (D = 0.73).

## Discussion

In the present study, we assessed the use of validated and new approaches for the differentiation of caprine MA isolates collected from restricted geographical areas with endemic CA. Data showed that although the number of isolates included in our study was limited, their genetic content was surprisingly diverse when compared to the previous situation reported for sheep [11,13,15]. Thus, typing tools already validated with MA mostly isolated from ovine, such as VNTR or PFGE assays [9,13,24], were shown to be also useful to discriminate MA collected in goats from a common geographical origin. This paves the way for addressing more complex epidemiological questions in the future. Our data also indicate that other molecular markers first used in this study would be worth evaluating on a larger scale, as they might increase the capacity to discriminate isolates with very similar profiles in PFGE or VNTR analyses. Where isolates cannot be distinguished by VNTR, the new assays offer alternative typing tools. Nevertheless, our data show that VNTR analyses using just VNTR17 is a powerful means of quickly discriminating goat MA isolates, even when they have been collected from a restricted endemic area.

The situation in Murcia (Spain) was ideal for this study because it has been known to have endemic CA for several decades [18,19] and the dominance of a native goat breed in almost all herds limits the entry of animals from other areas of Spain. Although a larger number of isolates now need to be typed, data collected thus far is already informative because of the congruence of all typing techniques. They point to the circulation of two clonal groups, one in Yecla (profile A) and one in Jumilla (profile B), each with distinctive molecular features. The isolates from Yecla resemble the PG2 type strain, while isolates from Jumilla are rich in mobile genetic elements that are not found in PG2. This suggests a different origin of infection with no transfer between these two areas, which are only 28 km apart (see Additional file 5: Figure S2). The two semen isolates from the Lorca area, 150 kms further South, gave almost identical profiles that differed from those collected from milk (in Lorca and other areas) as indicated by their *vpma* profiles and the presence of sequences coding for restriction modification systems (PCR set 4). Whether they represent clonal isolates is not known and there is certainly a need for detailed studies to determine whether isolates from semen form a true subgroup within MA. The prospect of associating a particular typing pattern with a particular tissue tropism is appealing but speculative at this time. To address this issue, more isolates collected in this area from semen and from milk or cases of mastitis would have to be analyzed.

In a recent study, a combination of genome-specific DNA probes and VNTRs indicated that CA in the Western Pyrenees region over the past 30 years has been caused by a unique subtype of MA [15]. This indicates that eradication programs implemented in the 1990s in this area, while efficient in reducing the prevalence of the disease, failed to eradicate the strain, which has re-emerged over the last few years, causing significant economic losses. The situation encountered in the endemic area in Spain differs in that the genetic diversity of the circulating strains is high and that several strains appear to be present despite the restricted sampling area. This situation is likely to be the result of a long period of colonization and evolution, but raises the question of whether it is a characteristic of MA in goats or whether it may also occur in sheep. In goats, clinical signs are rare, and sporadic outbreaks are often related to the introduction of infected animals from other herds [4]. It has been suggested that the genetic diversity of MA might be linked to the importation of animals, with MA in self-sufficient countries having less genetic heterogeneity [10]. In the endemic area of Murcia, importation of goats is not common practice and thus is unlikely to be the reason for the genetic diversity of MA. The occurrence of asymptomatic auricular carriers in goat herds, with several mycoplasma species cohabiting in the ear canal raises the prospect of genetic exchange, which may contribute to the genetic variation seen in the Spanish isolates.

## Conclusions

A number of typing tools are now available for the differentiation of goat MA isolates collected in restricted geographical areas with endemic CA. The genetic diversity of MA observed in infected goats in Spain contrasts with that seen in sheep with CA in southern France, suggesting that assessment of the disease status in endemic areas may require different approaches, depending on whether it involves goats or sheep.

## Methods

### Isolates and strains

A total of 13 field isolates were included in this study. They came from 3 main breeding areas (Jumilla, Yecla and Lorca) located in the goat-producing region of Murcia, Spain (see Additional file 5: Figure S2), a region known to have endemic CA [18,19]. They were isolated mainly from bulk tank milk (BTM) but two isolates were also obtained from the semen of 2 asymptomatic bucks (Table 1). One isolate from Málaga, a goat breeding area located further southwest, was also included. All these strains were analysed with strains PG2 and 5632, which were used as reference strains and have both been fully sequenced [16,17].

### Culture and DNA extraction

The isolates were identified as MA by both growth inhibition tests [25], dot immunoblotting on a filtration membrane (MF-dot) [26] and a species-specific PCR [11]. All strains were cultured in SP4 broth at 37°C [27]. Genomic DNA was extracted with chloroform using standard procedures [28].

### Pulsed field gel electrophoresis (PFGE)

Agarose plugs containing mycoplasma DNA were prepared as described previously [29]. They were incubated in 0.1% Triton X-100 for 2 hours at 4°C before digestion with restriction endonucleases overnight at 37°C using 50 units of either *Sma*I, *Mlu*I, *Bst*ZI and *Kpn*I (Promega, Charbonnières-les-Bains, France). Electrophoresis was performed using the CHEF-Mapper system (Bio-Rad, Marnes-la-Coquette, France) with the standard λ concatamer ladder (Bio-Rad) as a molecular size marker. Agarose gels (1%) were run in 0.5 X TBE at 14°C, at a field strength of 6 V/cm, over 24 h with an included angle of 120°, and the pulse time ramped linearly from 10 to 60 seconds. Gels were stained with ethidium bromide and visualized using the Gel-Doc 2000 image analysis system (Bio-Rad).

### Variable number of tandem repeats (VNTR)

VNTR analyses were performed as described previously [9,15]. Briefly, PCR assays were conducted in a Mastercycler ep-Gradient thermocycler (Eppendorf, Le Pecq, France) in a total volume of 25 μl containing 0.4 mM of each primer, 1 X PCR buffer (with MgSO_4_; New England Biolabs, Ipswich, MA, USA), 200 mM of each deoxynucleoside triphosphate and 2 units of *Taq* DNA polymerase (New England Biolabs). The PCR thermal program consisted of 5 min at 94°C, followed by 30 cycles of 1 min at 94°C, 45 s at 56°C and 1 min at 72°C, and a final extension step for 10 min at 72°C. The PCR amplification products were analysed by gel electrophoresis on 2.5% (w/v) agarose gels and visualised, after staining with ethidium bromide using, a UV transilluminator.

For each VNTR, one PCR product representative of each distinct profile was sequenced. The product obtained from the type strain PG2 was also sequenced. The PCR products were purified using the QIAquick PCR Purification kit (Qiagen, Alameda, CA, USA) and then sequenced by IF30, CHU Purpan, University of Toulouse (France). Tandem repeats in each sequence were identified using the on-line tandem repeat finder program at http://tandem.bu.edu/trf/trf.html. The data obtained were then compared using Needle at http://www.ebi.ac.uk/Tools/emboss/align/ and ClustalW2 at http://www.ebi.ac.uk/Tools/clustalw2/ to determine the consensus sequence for each VNTR, and the number of repeats in each product. Finally, strains were classified according to the size of the fragment and number of repeats.

### Southern blots

Several probes were used to examine genetic variability in strains of MA. The repertoires of *vpma* loci in the isolates were determined using 6 individual probes based on the PG2 sequence, and the general probe A3F [21,22]. Profiles were also obtained using a set of 7 AGA and BOV probes, previously shown to discriminate MA isolates from diverse origins [11]. In addition, the presence and number of ICEs in the genome was determined using Digoxigenin-labelled probes for ICEA_5632_ (CDS1, CDS5 and CDS22) [11,15,17]. Approximately 1 μg of genomic DNA was digested with *Hind*III, *Eco*RI (Promega Laboratories), *Ase*I or *Alw*I (New England Biolabs), subjected to 1% agarose gel electrophoresis and transferred onto nylon membranes (Roche, Indianapolis, IN, USA). Specific probes for each of the six *vpma* genes in PG2 (*vpmaU*, *vpmaV*, *vpmaW*, *vpmaX*, *vpmaY* and *vpmaZ*) were labelled with Digoxigenin-11-dUTP (Roche) by PCR, as described previously [21]. The probes AGA and BOV used in this study were prepared by PCR as described previously [11].

In all cases, after a denaturation step of 100°C, probes and membranes were incubated in Church buffer [30] overnight at 55°C. The membranes were then briefly washed in 0.2 x SSC/0.1% SDS at room temperature and then in 0.2 x SSC/0.1% SDS for 1 h at 55°C. The oligonucleotide probe A3F, (5Â´- AA(A/G)TG(T/C)GG(A/T)GG(A/T)AC(A/T)A(A/C)(A/T)(A/G)A-3Â´) [15,22] was incubated with the membranes overnight at 45°C and two washing steps were then conducted in 6 × SSC/0.1% SDS for 10 min each at 45°C. In all cases, hybridized probes were detected with alkaline phosphatase-conjugated anti-Dig Fab antibodies and CDPstar reagents (Roche).

### Identification of new PCR assays as typing tools

Several PCR assays (see Additional file 3: Table S3) were performed to explore differences between the strains on the basis of previously observed differences between PG2 and 5632 and on *in silico* comparison of their genomic se quences [16,17]. Further specific PCR assays were conducted. When necessary, primers were designed using the on-line program Primer3Plus at http://www.bioinformatics.nl/cgi-bin/primer3plus/primer3plus.cgi (see Additional file 6: Table S4). All PCRs were performed as described for the VNTR analysis by incubation for 2 min at 94°C, followed by 30 cycles of 30 s at 94°C/30 s at the appropriate melting temperature (see Additional file 3: Table S3) and 30 s at 72°C, and then by a final extension step of 5 min at 72°C. PCR products were analysed by electrophoresis in 1% agarose gels.

## Competing interests

The authors declare that they have no competing interests.

## Authors’ contributions

CDF conceived the study, carried out part of the genomic studies, participated in its design and drafted the manuscript. JA carried out the PFGE genomic studies and drafted the manuscript. FT participated in the design and the coordination of the study, contributed to PFGE studies and drafted the manuscript. ES carried out part of the genomic studies. LXN participated in study design and coordination, and drafted the manuscript. CC conceived the study, participated in its design and coordination, and drafted the manuscript. All authors read and approved the manuscript.

## Supplementary Material

Additional file 1 **Table S1.** VNTR nucleotide sequences. Click here for file

Additional file 2 **Table S2.** Profiles obtained with the probe A3F, the set of individual Vpma probes and the AGA-BOV probes used in this study. Click here for file

Additional file 3 **Table S3.** Description of additional PCRs conducted in this study, the results of which are summarized in Table 1. Click here for file

Additional file 4 **Figure S1.** Illustration of some genomic differences between M. agalactiae strains PG2 and 5632 that were used to design new PCR typing assays. Click here for file

Additional file 5 **Figure S2.** Geographic origin of Mycoplasma agalactiae isolates. Click here for file

Additional file 6 **Table S4.** Primer sequences. Click here for file
