# Supplementary material for: Unexpected genetic diversity of Mycoplasma agalactiae caprine isolates from an endemic geographically restricted area of Spain
Source: BMC Vet Res. 2012 Aug 27;8:146. doi: 10.1186/1746-6148-8-146 (PMC3514313; doi:10.1186/1746-6148-8-146)
Supplement: Additional file 1 — Table S1. VNTR nucleotide sequences. [file 1746-6148-8-146-S1.pdf]

Table S1: VNTR nucleotide sequences

VNTR/ Profile	Strains	Nº of repetitions	Sequences
**VNTR 5**	**AG13**	**2.1**	GAAAGAGAAAGGAAGCTGAAGCAGAGAAAGCCTAAGAAAAATGTAGAGGAATTTAGGAAGCTGTTTAAAGAATTAGAAAAAATTAAAAAAGAATATGATGAAGTAAAAAGCAGATTAACTGGGCCTGAATATGAAACCATAAAGACTAAATATGAAAGTTTCTTTTATGCAAGTGGATACAATCAATACACACAATTTATGGAGCAATCAAAAAGCGATAATGATCC
**VNTR 5**	**PG2**	**2.1**	GAAAGAGAAAGGAAGCTGAAGCAGAGAAAGCCAAGAAAAATGTAGAGGAATTTAGGAAGCTGTTTAAAGAATTAGAAAAAATTAAAAAAGAATATGATGAAGTAAAAAGCAGATTAACTGGGCCTGAATATGAAACCATAAAGACTAAATATGAAAGTTTCTTTTATGCAAGTGGATACAATCAATACACACAATTTATGGAGCAATCAAAAAGCGATAATGATCC
**VNTR 14**	**AG 4**	**2**	TTGAAATATCCGCTTAAGAAAAAAGTAATTAATAATTATATATTTTATACTGTTTTAGATTGCTAATTTAGATTGCTAAATAATGACACTAAATCAGTTGATGATAAACTATATTGACTCTCTGTGTCAATTACTATTAGCACCATTAAATGCAAATT
**VNTR 14**	**PG2**	**2**	TTGAAATATCCGCTTAAGAAAAAAGTAATTAATAATTATATATTTTATACTGTTTTAGATTGCTAATTTAGATTGCTAAATAATGACACTAAATCAGTTGATGATAAACTATATTGACTCTCTGTGTCAATTACTATTAGCACCATTAAATGCAAATT
**VNTR 17**	**5632**	**6.4**	TTTAGCTTTTGATTCAATACTTTCTAGTATCTTATTTTTCATATTTACCTTTTATTTTGTTTCTGTTTATTACCTTTTATATACCTTCTAGTTATTACCTTTATTAGTTGTCTTTTATATACCTTCTATAACCTTCTAGTTTTTATTACCTTTTATATACCTTCTAGTTACTGTCTTTATTAGTTGTCTTTTATATACCTTCTATAACCTTCTAGTTTTTATTACCTTTTATATACCTTCTAGTTATTGTCTTTCATATACCTTCTATAACCTTCTAGTTGATGATTGAATTTATTAAAATTCTCTTATTATCTAGTTTTAAAGAACTAAATGCTCGCATAATTCTTT
**VNTR 17**	**AG 32**	**6.3**	TTTAGCTTTTTGATTCAATACTTTCTAGTATCTTATTTTTCATATTTACCTTTCATATGCCTTCTAGTTATTACCTTTATTAGTTGTCTTTTATATACCTTCTATAACCTTCTAGTTTTTATTACCCTTCATATACCTTCTAGTTATTACCTTTATTAGTTGTCTTTTATATACCTTCTATAACCTTCTAGTTTTTATTACCTTTTATATACCTTCTAGTTATTACCTTTATTGGTTGTCTTTTATATACCTTCTATAACCTTCTAGTAGATAATTCAATTTATTAAAAATCTCTTATTATCTAGTTTTAAAAATCTAAATGCTCGCATAATTCTTT

**VNTR 17**	**AG 30**	**4.4**	TTTAGCTTTTGATTCAATACTTTCTAGTATCTTATTTTTCATATTTACCTTTTATTTTGTTTCTATTTATTAC<br>CTTTTATATACCTTCTAGTTATTACCTTTATTAGTTGTCTTTTATATACCTTCTATTACCTTCTAGTTTTCAT<br>TACCTTTTATATACCTTCTAGTTATTGCCTTTATTAGTTGTCTTTTATATACCTTCTATTACCTTCAAGTAG<br>ATAATTCAATTTATAAAAAATCTCTTATTATCTAGTTTTAAAGATCTAAATGCTCGCATAATTCTTT
**VNTR 17**	**PG 2**	**2.6**	TTTAGCTTTTGATTCAATACTTTCTAGTATCTTATTTTTCATATTTACCTTTTATTTTGTTTCTATTTATTAC<br>CTTTTATATACCTTCTAGTTATTACCTTTATTAGTTGTCTTTTATATACCTTCTATTACCTTCAAGTAGATA<br>ATTCAATTTATTAAAAATCTCTTATTATCTAGTTTTAAAGATCTAAATGCTCGCATAATTCTTT
**VNTR 19**	**AG 14**	**4.9**	TTGCTTCTTGTGCTTCTTTTTTCTTTCTAGCTGCTTCTTCTTGCTTTCTTTTCTCTTCTTTTTGTTTTCTTTCCG<br>CTTCTTCTTTTGCTTTAGCAGCCTCTTCTTGTATTCTTTTTGTTTCCTCTATTGTATGCGGCATAGCATATGT<br>GCCACCATTATTTTCTGAATTATCTGGTTGATCCCCTT
**VNTR 19**	**PG 2**	**4.9**	TTGCTTCTTGTGCTTCTTTTTGTTTCCTTGTAGCTTCTTCTTGCTTTCTTTTTTTCTTCTTGTTGTTTTCTTTCT<br>TCTTCTTTTGCTTTAGTGGCCTCTTCTTGTATTCTTTTTGCTTCCTCTAATGTATGCGGCATAGCATATGTG<br>CCACCATTATTTTCTGAATTATCTGGTTGATCCCCTT
