# Supplementary material for: Unexpected genetic diversity of Mycoplasma agalactiae caprine isolates from an endemic geographically restricted area of Spain
Source: BMC Vet Res. 2012 Aug 27;8:146. doi: 10.1186/1746-6148-8-146 (PMC3514313; doi:10.1186/1746-6148-8-146)
Supplement: Additional file 2 — Table S2. Profiles obtained with the probe A3F, the set of individual Vpma probes and the AGA-BOV probes used in this study. [file 1746-6148-8-146-S2.pdf]

**Table S2**: Southern blot profiles obtained with the probe A3F, the set of individual *vpma* probes and the AGA-BOV probes used in this study.

| Strain | A3F probe | Individual *vpma* probes | | | | | | *vpma* profile | AGA (A) and BOV (B) probes | | | | | | | AGA/BOV profile |
|---|---|---|---|---|---|---|---|---|---|---|---|---|---|---|---|---|
| | Nb of bands [bands size (kb)] [a] | U | V | W | X | Y | Z | | A27 | A49 | A79 | B4 | B15 | B64 | B88 | |
| PG2 | 4 [2.4/1.4/1.1/0.6] | 1 [2.4] | 1 [1.1] | 3 [1.1/0.6/0.3] | 1 [1.1] | 1 [1.4] | 1 [2.4] | VP1a | 1 [1.7] | 1 [2.0] | 2 [3.6/1.5] | 0 | 0 | 1 [1.2] | 0 | AGB1 |
| AG30 | 5 [2.6/1.4/1.1/1.2[b]/0.6] | 1 [2.6] | 1 [1.1] | 4 [1.1/0.6/0.3/1.5] | 1 [1.1] | 1 [1.4] | 1 [2.6] | VP2 | 1 [1.7] | 1 [2.0] | 2 [3.6/1.5] | 0 | 0 | 1 [1.2] | 0 | AGB1 |
| AG33 | 5 [2.6/1.4/1.1/1.2[b]/0.6] | 1 [2.6] | 1 [1.1] | 4 [1.1/0.6/0.3/1.5] | 1 [1.1] | 1 [1.4] | 1 [2.6] | VP2 | 1 [1.7] | 1 [2.0] | 2 [3.6/1.5] | 0 | 0 | 1 [1.2] | 0 | AGB1 |
| AG35 | 5 [2.6/1.4/1.1/1.2[b]/0.6] | 1 [2.6] | 1 [1.1] | 4 [1.1/0.6/0.3/1.5] | 1 [1.1] | 1 [1.4] | 1 [2.6] | VP2 | 1 [1.7] | 1 [2.0] | 2 [3.6/1.5] | 0 | 0 | 1 [1.2] | 0 | AGB1 |
| AG28 | 5 [2.6/1.4/1.1/1.2[b]/0.6] | 1 [2.6] | 1 [1.1] | 4 [1.1/0.6/0.3/1.5] | 1 [1.1] | 1 [1.4] | 1 [2.6] | VP2 | 1 [1.7] | 1 [2.0] | 2 [3.6/1.5] | 0 | 0 | 1 [1.2] | 0 | AGB1 |
| AG18 | 5 [2.4/1.4/1.1/ 1.2[b]/0.6] | 1 [2.4] | 1 [1.1] | 4 [1.1/0.6/0.3/1.5] | 1 [1.1] | 1 [1.4] | 1 [2.4] | VP1b | 1 [1.7] | 1 [2.0] | 2 [3.6/1.5] | 0 | 0 | 1 [1.2] | 0 | AGB1 |
| AG4 | 5 [2.6/1.4/1.1/1.2[b]/0.6] | 1 [2.6] | 1 [1.1] | 4 [1.1/0.6/0.3/1.5] | 1 [1.1] | 1 [1.4] | 1 [2.6] | VP2 | 1 [1.7] | 1 [2.0] | 2 [3.6/1.5] | 0 | 0 | 1 [1.2] | 0 | AGB1 |
| AG13 | 5 [2.1[b]/1.9/1.8[b]/1.5[b]/0.9[b]] | 0 | 0 | 3 [1.3/0.6/0.3] | 1 [1.3] | 0 | 0 | VP3a | 0 | 1 [1.1] | 2 [3.6/1.5] | 0 | 0 | 2 [2.7/1.2] | 1 [3.9] | AGB2 |
| AG14 | 5 [1.5[b]/1.2[b]/1.1[b]/0.9[b]/0.8[b]] | 0 | 0 | 3 [1.3/0.6/0.3] | 1 [1.3] | 0 | 0 | VP3b | 0 | 1 [1.1] | 2 [3.6/1.5] | 0 | 0 | 2 [2.7/1.2] | 1 [3.9] | AGB2 |
| AG26 | 7 [2.3/2.0/1.8/1.4[b]/1.1[b]/0.6/0.3] | 1 [1.8] | 2 [2.0/1.8] | 4 [2.0/1.8/0.6/0.3] | 1 [1.9] | 0 | 3 [2.3/2.0/1.8] | VP5 | 0 | 2 [2.0/1.1] | 2 [3.6/1.5] | 0 | 2 [3.2/1.9] | 1 [1.2] | 2 [3.9/3.5] | AGB3 |
| AG27 | 7 [2.3/2.0/1.8/1.4[b]/1.1[b]/0.6/0.3] | 1 [1.8] | 2 [2.0/1.8] | 4 [2.0/1.8/0.6/0.3] | 1 [1.9] | 0 | 3 [2.3/2.0/1.8] | VP5 | 0 | 2 [2.0/1.1] | 2 [3.6/1.5] | 0 | 2 [3.2/1.9] | 1 [1.2] | 2 [3.9/3.5] | AGB3 |
| AG29 | 7 [2.3/2.0/1.8/1.4[b]/1.1[b]/0.6/0.3] | 1 [1.8] | 2 [2.0/1.8] | 4 [2.0/1.8/0.6/0.3] | 1 [1.9] | 0 | 3 [2.3/2.0/1.8] | VP5 | 0 | 2 [2.0/1.1] | 2 [3.6/1.5] | 0 | 2 [3.2/1.9] | 1 [1.2] | 2 [3.9/3.5] | AGB3 |
| AG32 | 7 [2.3/2.0/1.8/1.4[b]/1.1[b]/0.6/0.3] | 1 [1.8] | 2 [2.0/1.8] | 4 [2.0/1.8/0.6/0.3] | 1 [1.9] | 0 | 3 [2.3/2.0/1.8] | VP5 | 0 | 2 [2.0/1.1] | 2 [3.6/1.5] | 0 | 2 [3.2/1.9] | 1 [1.2] | 2 [3.9/3.5] | AGB3 |
| AG34 | 7 [2.3/2.0/1.8/1.4[b]/1.1[b]/0.6/0.3] | 1 [1.8] | 2 [2.0/1.8] | 4 [2.0/1.8/0.6/0.3] | 1 [1.9] | 0 | 3 [2.3/2.0/1.8] | VP5 | 0 | 2 [2.0/1.1] | 2 [3.6/1.5] | 0 | 2 [3.2/1.9] | 1 [1.2] | 2 [3.9/3.5] | AGB3 |
| 5632 | 4 [1.7[b]/1.4/1.0[b]/0.8[b]] | 0 | 0 | 0 | 0 | 1 [1.4] | 1 [1.4] | VP7 | 0 | 1 [1.1] | 2 [3.6/0.8] | 0 | 0 | 2 [2.1/1.2] | 2 [2.6/2.1] | AGB4 |

[a] Number of bands recognized is indicated for each probe used, with estimated band size in brackets.
[b] Bands revealed by A3F hybridization but not by individual *vpma* probes
