# Supplementary material for: Unexpected genetic diversity of Mycoplasma agalactiae caprine isolates from an endemic geographically restricted area of Spain
Source: BMC Vet Res. 2012 Aug 27;8:146. doi: 10.1186/1746-6148-8-146 (PMC3514313; doi:10.1186/1746-6148-8-146)
Supplement: Additional file 3 — Table S3. Description of additional PCRs conducted in this study, the results of which are summarized in Table 1. [file 1746-6148-8-146-S3.pdf]

Table S3: Description of additional PCR assays conducted in this study, the results of which are summarized in Table 1

PCR set	Targeted gene/ position	Mnemonic PG2 / 5632[a]	Primer pair	PCR product size (bp) expected in PG2 / 5632	Melting T[a]	PCR designed
Control	*p48* (Lipoprotein)	MAG0120 / MAGa0140	P48F / P48R	416/416	50ºC	In this study
Control	*polC*	*polC*/*polC*	MAPol-1F/ MAPol-5R	265/265	49ºC	*Microbiology* 2005
1	IS*Mag1* position	- / MAGa8240	xerF / phydR	932 / 2463	55ºC	*J.Bact.* 2009
1	IS*Mag1* position	- / MAGa5890	xerF / agpR	0 / 1890	55ºC	*J.Bact.* 2009
1	IS*Mag1* position	- / MAGa5790	Mag2F / pv1R	0 / 2233	55ºC	*J.Bact.* 2009
1	IS*Mag1* position	- / -	Mag2F / agpR	608 / 0	55ºC	*J.Bact.* 2009
1	IS*Mag1* position	- / MAGa5330	pks1F / pks1R2	1084 / 2614	50ºC	In this study (detail in Figure 2S panel C)
2	IS*Mag1* position	- / MAGa4500	Bsp6IF / Bsp6IR_5632	3201 / 4512	50°C	In this study (detail in Figure 2S panel B)
3	IS*Mag1*	- / all IS*Mag1*[b]	ISMag1F2 / ISMag1R2	0 / 970	55°C	In this study
3	IS*Mag2*	- / MAGa1590	IS30L / IS30LR	0 / 983	59ºC	In this study
3	*cds1*	- / MAGa2970	cds1F / cds1R	0 / 410	50ºC	*J Bact* 2006
3	*cds5*	- / MAGa3060	cds5F-cds5R	0 / 214	50ºC	In this study
3	*cds22*	- / MAG3220	cds22F / cds22R	0 / 401	50ºC	*J Bact* 2006
3	*bsp6I methylase*	- / MAGa4250	bsp6ImR / bsp6ImF	0 / 505	59°C	In this study
4	*DNA methylase*	- / MAGa2700	Met1F/ Met1R5632	0 / 169	50°C	In this study (detail in Figure 3 panel A)
4	*cdsH*	- / MAGa3200,5050,6900	cdshF / cdshR	0 / 208	57ºC	In this study
5	*abiGI-like*	- / MAGa8140	aip1F / aip1R	0 / 358	55°C	*J Bact* 2009
5	*abiGI-like and abiGII-like*	- / MAGa8140 and MAGa8130	aip2F / aip2R	0 / 1011	55°C	*J Bact* 2009
5	*fic*	- / MAGa7460	ficF5632 / ficR	0 / 398	50ºC	In this study
5	*dcm*	- / MAGa3950	dcmF / dcmR	0 / 653	59ºC	In this study
6	*DNA methylase pseudogene*	MAG2550-2560 / -	Met1F/ Met1RpG2	395 / 0	50°C	In this study (detail in Figure 3 panel A)
6	*Type III DNA methylase*	MAG1530	Met3FPg2 / tigR	2268 / 0	50ºC	In this study

[a] Mnemonic for CDS of *Mycoplasma agalactiae* strains PG2 and 5632 according to MolliGen database at http://cbi.labri.fr/outils/molligen/
[b] PCR primers matching all 12 copies of IS*Mag1* in 5632 genome.
