# Supplementary material for: Unexpected genetic diversity of Mycoplasma agalactiae caprine isolates from an endemic geographically restricted area of Spain
Source: BMC Vet Res. 2012 Aug 27;8:146. doi: 10.1186/1746-6148-8-146 (PMC3514313; doi:10.1186/1746-6148-8-146)
Supplement: Additional file 4 — Figure S1. Illustration of some genomic differences between M. agalactiae strains PG2 and 5632 that were used to design new PCR typing assays. [file 1746-6148-8-146-S4.pdf]

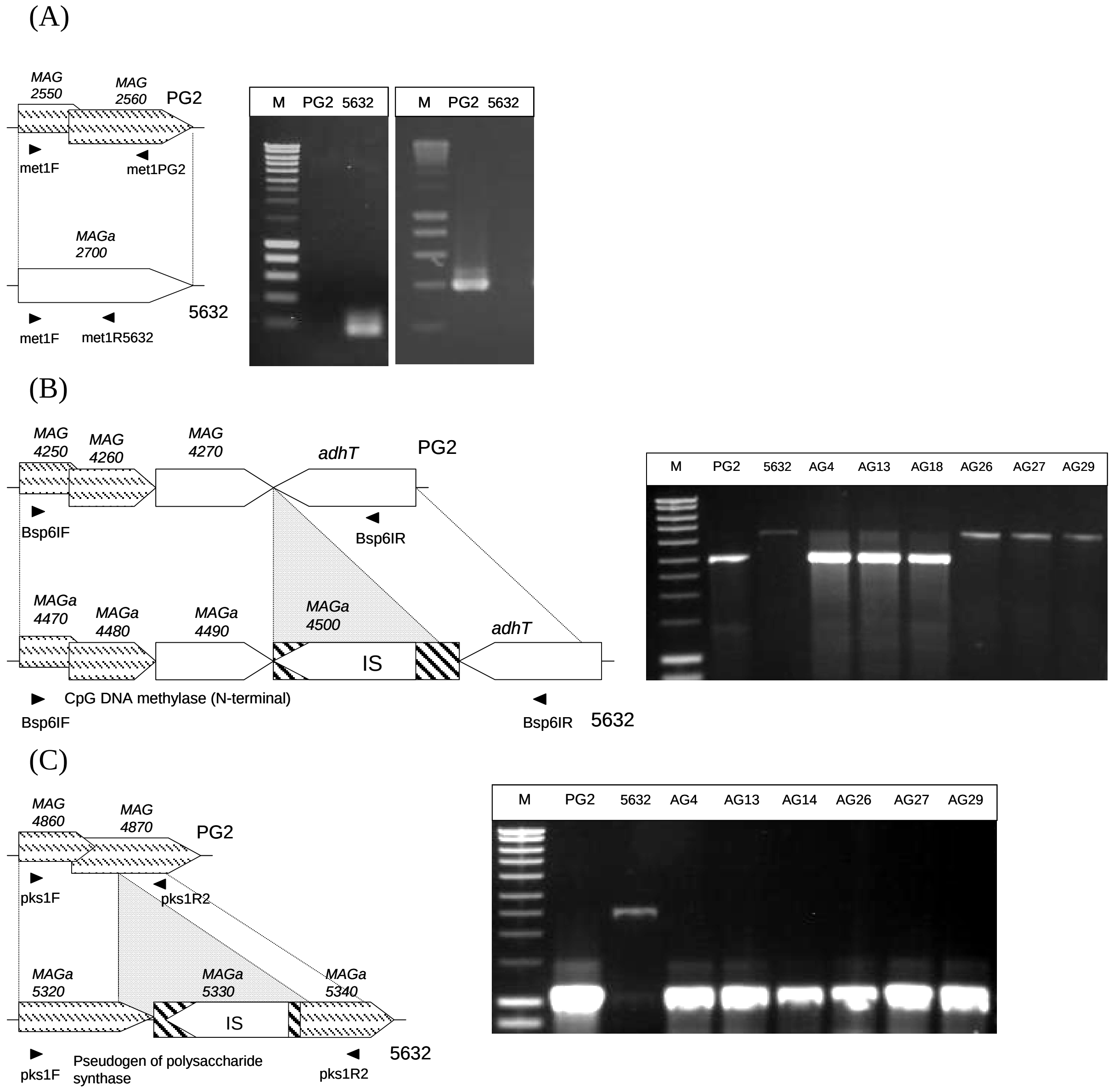

Figure S1: Illustration of some genomic differences between *Mycoplasma agalactiae* strains PG2 and 5632 that were used to design new PCR typing assays.

Each panel on the left shows the gene organization in the targeted region and on the right the corresponding PCR results. Open arrows correspond to CDSs of strain PG2 (mnemonic “MAG”) or of strain 5632 (mnemonic “MAGa”). Small arrow heads represent PCR primers used to generate amplicons shown on the right side of each panel. Open arrows filled with a pattern represent pseudogenes or genes that are disrupted by an Insertion Sequence (IS) element. The molecular weight markers (Smart Ladder Eurogentec) are also indicated (M).
