# Supplementary figures and images for: Unexpected genetic diversity of Mycoplasma agalactiae caprine isolates from an endemic geographically restricted area of Spain
Source: BMC Vet Res. 2012 Aug 27;8:146. doi: 10.1186/1746-6148-8-146 (PMC3514313; doi:10.1186/1746-6148-8-146)

**Figure S2:** Geographic location of *M. agalactiae* isolates.

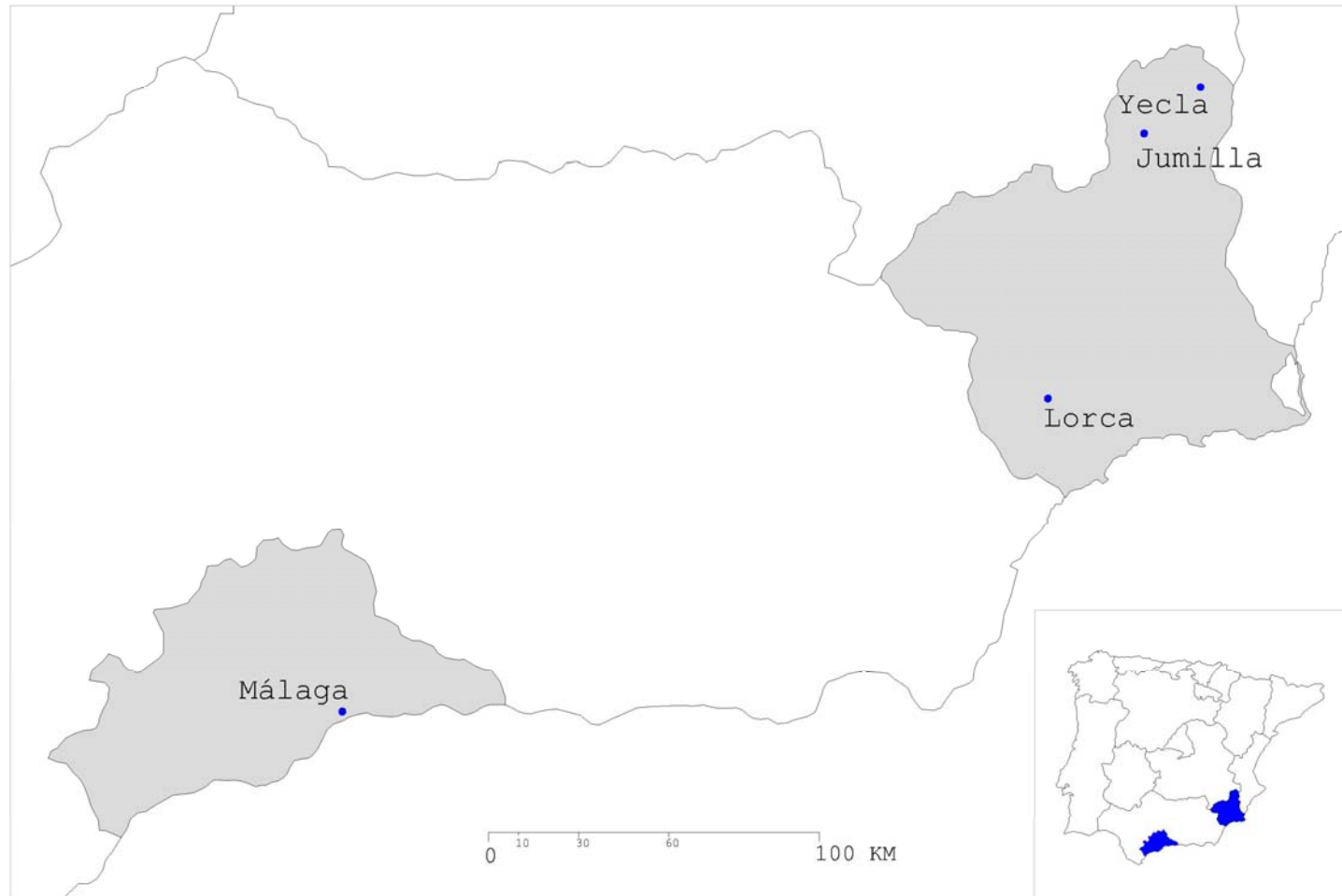

Supplement: Additional file 5 — Figure S2. Geographic origin of Mycoplasma agalactiae isolates. [file 1746-6148-8-146-S5.pdf]
