# Supplementary material for: Unexpected genetic diversity of Mycoplasma agalactiae caprine isolates from an endemic geographically restricted area of Spain
Source: BMC Vet Res. 2012 Aug 27;8:146. doi: 10.1186/1746-6148-8-146 (PMC3514313; doi:10.1186/1746-6148-8-146)
Supplement: Additional file 6 — Table S4. Primer sequences. [file 1746-6148-8-146-S6.pdf]

Table S4: Primer sequences

Name	Sequence (5’ to 3’)
5F	GAAAGAGAAAGGAAGCTGAA
5R	GGATCATTATCGCTTTTTGA
14F	TTGAAATATCCGCTTAAGAAA
14R	AATTTGCATTTAATGGTGCT
17F	TTTAGCTTTTGATTCAATACTTTC
17R	AAAGAATTATGCGAGCATTT
19F	TTGCTTCTTGTGCTTCTTTT
19R	AAGGGGATCAACCAGATAAT
MAPol-1F	CATTGAACCTCTTATGTCATTTACTTTG
MAPol-5R	CTATGTCATCAGCTTTTGGGTGA
phydR	TCAGGAATTGAGTGCTGTGC
xerF	TGCGTAGATCATTGCTTCG
pv1R	TCTGCCACTGATTACAACTTATGAA
agpR	TCAAAAGGATATTTTACACCATGTC
Mag2F	CTCCGATCTTCTAGCGAAGC
aip1F	TTGCTTTTGTGCTTAAATACTTGT
aip1R	TATAGCCACTGTAAACCGTAAC
aip2F	ATGCAAATGTTTACTACTGTGATAG
aip2R	TCATCTTTCATTCATCAACGGATT
ISMag1F2	TGATTGATGAAACACATTGAGATG
ISMag1R2	GATGAATAAGGGTGGCAATG
bsp6ImR	GTCCCCATATTGGCTGTTAAAG
bsp6ImF	CTGGCTATCGTAAAGGGTTCG
dcmF	GACGCATGTCAAACGCTAAG
dcmR	CAAAATTCCAGTTCTGCCACC
IS30L	GTTCCACACTTGGCAGCAG
IS30L-R2	CTTTTGCCTATAATTAAGTCGC
cds1F	CCAGTTGTTACACTTGGCTTTTT
cds1R	CAAAGGGTTGTTGCTGATCC
cds5F	TGGGATGCAAATCAAGACTG
cds5R	CAAAGCTTTTTGTTTGTGCAAT
cds22F	TGAGACCAGCAAGCTGAAGA
cds22R	TCTGTATCAATCTGAATTGCATCAT
Met1F	TAAGCGCTGTTTCAATGGTC
Met1-R_5632	GTTTGAACTGTTATTTCGAAG
Met1-R_Pg2	CTTTTATGACTGTGATATAGTAA
cdsH-F	GCGGTTCAGGAATAGTTGGA
cdsH-R	ATGTTTAGTTTATCCAATCAGCC
Bsp6IF	TTATTTGCTGGTATTGGAAGC
Bsp6I-R_5632	GCACTTCTAGAGTCGTAACTTC
Met3-F_Pg2	GATTTGACCATCAGGTGCTTT

tig-R	AAATGCATTGCAAAATGCTG
p48-F	GCAGCTTGTTTAGTGTCAAAG
p48-R	CCTAAAGCAACCTTTATAACTG
fic-F_5632	GCTGACACTAAGAGAAACAGTG
fic-R	CTACTTGTTCTACCATTACCATC
pks1-F	AGAAGGCTTTAAGGCCGGTA
pks1-R2	GCCTTTGGTTTCTGTTTTGC
